# Supplementary material for: Lack of drug-induced post-retrieval amnesia for auditory fear memories in rats
Source: BMC Biol. 2021 Jan 26;19:17. doi: 10.1186/s12915-021-00957-x (PMC7836479; doi:10.1186/s12915-021-00957-x)
Supplement: Supplementary file 1 — Additional file 1. Contains details about any adverse reactions to the applied drugs. [file 12915_2021_957_MOESM1_ESM.pdf]

Additional file 1

Adverse reactions to the applied drugs

As shown in the main article (***Fig. 2E*** and ***Fig. 3C***), rapamycin, anisomycin and cycloheximide had long-term effects on body weight, with lower weights in drug-treated animals than controls several days after injection.

Furthermore, we observed an increase in freezing(-like) behavior during the short-term memory test in Experiment 7 (***Fig. 3B*** and ***Table 2***). Freezing was scored by an observer blinded to group allocation, but it seems that acute (i.e., 4 hours after injection) side effects may have confounded the freezing measurements during this particular test (see below). Whether these effects have a sedative, numbing, nauseous or other nature is impossible to say, but we can assume that, to a certain degree, the resulting immobility was mistakenly identified as freezing. This is especially clear during the PreCS measurement on Test STM (***Fig. 3B***), where vehicle animals show very little freezing (8% on average), whereas animals that had received a protein synthesis inhibitor 4 hours earlier, were classified as more immobile, anisomycin animals (66%) even to a greater extent than cycloheximide animals (43%). During the CS presentations, the differences are much smaller, presumably due to a ceiling effect (high freezing in vehicle animals as well). Do note that these effects had disappeared by the next day (PreCS freezing on Test 1), suggesting that the animals had recovered sufficiently and that the disruptive acute effects that were seen a few hours after injection were no longer relevant 20 hours later, implying a reliable behavioral read-out for all animals on Test 1 and 2. This was confirmed by the absence of effects on contextual freezing in Test 1 and Test 2 of Experiment 8 as well (***Fig. 3E, G***).

In addition to analyzing the acute effects that were just described, we carried out a qualitative assessment of the animals' wellbeing in the hours and days after injection, given the potential toxic effects of systemic drugs that are intended to interfere with protein synthesis. Manufacturers' safety sheets (based on the Registry of Toxic Effects of Chemical Substances) and the literature provided the following toxicity information: LD_{50} DMSO (intraperitoneal, rat): 9.9 ml/kg (Bartsch et al. 1976), LD_{50} rapamycin (intraperitoneal, rat): 18.2 mg/kg; LD_{50} anisomycin (subcutaneous, rat): 230 mg/kg; LD_{50} cycloheximide (subcutaneous, rat): 2.5 mg/kg. Note that the supposed LD_{50} of rapamycin is rather unexpected, given our own and others' experiences with this drug at doses of 20-40 mg/kg (Hoffman et al. 2015; Li et al. 2013; Tallot et al. 2017). Adverse reactions to systemic administration of the protein synthesis inhibitors have only rarely been reported in prior publications. A few did mention that 150 mg/kg anisomycin produced signs of distress (e.g., lethargy, balance problems, piloerection, hunched back posture) in the hours after injection and weight loss in the longer run (Blaiss & Janak, 2007; Hernandez & Kelley, 2004). Gisquet-Verrier et al. (2015) reported that two rats died after an intraperitoneal injection with 2.8 mg/kg cycloheximide (LD_{50}: 3.7 mg/kg).

In our own studies, we made qualitative assessments that were not embedded in the preregistered experimental design, but which were all observations made in the course of the experiment, by a researcher blinded to group division.

Rapamycin at a dose of 20 mg/kg (Experiment 5) did not seem to elicit important adverse reactions, apart from the above-mentioned reduced body weight that was seen with both applied doses. In

Experiment 6, where we used 40 mg/kg rapamycin, 4 out of 8 drug-treated animals presented with light and two with more pronounced diarrhea one day after injection. Note that, in addition, two out of 8 vehicle animals had light diarrhea, suggesting that the gastrointestinal effects may be ascribed mainly to rapamycin, but partly also to the DMSO vehicle. On the following days, no adverse reactions were noted.

In Experiment 7, all animals were responsive and showed normal muscular tonus when taken out the home cage for the short-term memory test, 4 hours after injection. Nevertheless, careful observation of the animals in the hours after injection did indicate slight to pronounced orbital tightening in 9 out of 10 **anisomycin**-treated rats. Eight of these also showed diarrhea, and two of them were even briefly lying on their back when checked about one hour after injection. Note that all animals seemed to have recovered sufficiently by the next day, and no behavioral abnormalities, nor signs of pain or distress were observed on the day of Test 1. Nine anisomycin animals did show slight skin irritation at the site of injection when examined after euthanasia. Three out of 14 **cycloheximide**-treated rats showed orbital tightening in the hours after injection, and one of these animals also had diarrhea. All animals seemed to have recovered sufficiently by the next day (Test 1 session). Finally, as described above, both anisomycin and cycloheximide injections resulted in lower body weight gain.

References

Bartsch, W., Sponer, G., Dietmann, K., & Fuchs, G. (1976). Acute toxicity of various solvents in the mouse and rat. LD50 of ethanol, diethylacetamide, dimethylformamide, dimethylsulfoxide, glycerine, N-methylpyrrolidone, polyethylene glycol 400, 1,2-propanediol and Tween 20. *Arzneimittel-Forschung*, 26(8), 1581-1583.

Blaiss, C. A., & Janak, P. H. (2007). Post-training, but not post-reactivation, administration of amphetamine and anisomycin modulates Pavlovian conditioned approach. *Neurobiology of Learning and Memory*, 87(4), 644-658. doi: 10.1016/j.nlm.2006.12.007

Gisquet-Verrier, P., Lynch, J. F., 3rd, Cutolo, P., Toledano, D., Ulmen, A., Jasnow, A. M., & Riccio, D. C. (2015). Integration of New Information with Active Memory Accounts for Retrograde Amnesia: A Challenge to the Consolidation/Reconsolidation Hypothesis? *Journal of Neuroscience, 35*(33), 11623-11633. doi: 10.1523/jneurosci.1386-15.2015

Hernandez, P. J., & Kelley, A. E. (2004). Long-term memory for instrumental responses does not undergo protein synthesis-dependent reconsolidation upon retrieval. *Learning & Memory, 11*(6), 748-754. doi: 10.1101/lm.84904

Hoffman, A. N., Parga, A., Paode, P. R., Watterson, L. R., Nikulina, E. M., Hammer, R. P., Jr., & Conrad, C. D. (2015). Chronic stress enhanced fear memories are associated with increased amygdala zif268 mRNA expression and are resistant to reconsolidation. *Neurobiology of Learning and Memory, 120*, 61-68. doi: 10.1016/j.nlm.2015.02.004

Li, Y., Meloni, E. G., Carlezon, W. A., Jr., Milad, M. R., Pitman, R. K., Nader, K., & Bolshakov, V. Y. (2013). Learning and reconsolidation implicate different synaptic mechanisms. *Proceedings of the National Academy of Sciences of the United States of America, 110*(12), 4798-4803. doi: 10.1073/pnas.1217878110

Tallot, L., Diaz-Mataix, L., Perry, R. E., Wood, K., LeDoux, J. E., Mouly, A. M., Sullivan, R. M., & Doyere, V. (2017). Updating of aversive memories after temporal error detection is differentially modulated by mTOR across development. *Learning & Memory, 24*(3), 115-122. doi: 10.1101/lm.043083.116
