# Supplementary material for: Lack of drug-induced post-retrieval amnesia for auditory fear memories in rats
Source: BMC Biol. 2021 Jan 26;19:17. doi: 10.1186/s12915-021-00957-x (PMC7836479; doi:10.1186/s12915-021-00957-x)
Supplement: Supplementary file 2 — Additional file 2. Contains a supplementary discussion regarding indices of destabilization. [file 12915_2021_957_MOESM2_ESM.pdf]

Additional file 2

Supplementary discussion regarding indices of destabilization

As mentioned in the main text, we cannot exclude that we failed to destabilize the tone fear memory, which is thought to be a requirement for inducing amnesia. The only possible way to compare our behavioral procedures with those of the publications that they were based upon, is to look at the freezing levels, as an indication of how well we reproduced the behavior in the control condition. In Experiment 3, which was an exact replication attempt of (Debiec & LeDoux, 2004), mean freezing levels during the Reactivation CS and Test 1 (average of CS1-4) in the control condition were very similar in our study (67% and 57%, respectively) and theirs (75% and 67%, respectively, values obtained from graphs), suggesting that the protocol produced comparable levels of fear in our hands. Similar observations can be made for Experiment 7, where the behavioral procedure was an exact replication of Duvarci et al. (2005) and Nader et al. (2000). In other words, our exact replications, and the same holds for our other experiments, showed freezing levels that were comparable to those of successful studies in the literature, suggesting clear retrieval of the fear memory and sufficient room to observe amnesia at test.

Unfortunately, freezing may not suffice as an index of reactivation (Ben Mamou et al. 2006). Given the limitations of behavioral read-outs, prior research has looked for biomarkers that may indicate whether a memory is susceptible to post-retrieval modification. Although such markers currently have little translational value, they may offer insights into what is going on in our animals' brains and may even help to optimize procedural parameters for destabilization. Some have tried to estimate the probability that the memory will undergo destabilization, e.g., by quantifying NR2B NMDA-receptor subunits in the basal and lateral amygdala after training (Wang et al. 2009). Others have looked at plasticity during the reactivation session and found changes in the expression of the immediate early gene zif-268 in the lateral amygdala (Diaz-Mataix et al. 2013) or in the amount of polyubiquitination (a marker of protein degradation) in the entire amygdala (Jarome et al. 2011). However interesting, in order for such biomarkers to be informative, one in principle requires a comparison group that does show post-retrieval amnesia. Given that this was never the case in our hands, biomarker detection would not have been helpful in delineating adequate reactivation conditions. Moreover, we should not overestimate the decisive value of such markers and keep in mind that they are likely dependent on the behavioral procedure that is being used, the brain region under investigation and perhaps even prior experience of the subject (Finnie & Nader, 2012; Reichelt & Lee, 2013).

References

Ben Mamou, C., Gamache, K., & Nader, K. (2006). NMDA receptors are critical for unleashing consolidated auditory fear memories. *Nature Neuroscience, 9*(10), 1237-1239. doi: 10.1038/nn1778

Debiec, J., & LeDoux, J. E. (2004). Disruption of reconsolidation but not consolidation of auditory fear conditioning by noradrenergic blockade in the amygdala. *Neuroscience, 129*(2), 267-272. doi: 10.1016/j.neuroscience.2004.08.018

Diaz-Mataix, L., Ruiz Martinez, R. C., Schafe, G. E., LeDoux, J. E., & Doyere, V. (2013). Detection of a temporal error triggers reconsolidation of amygdala-dependent memories. *Current Biology, 23*(6), 467-472. doi: 10.1016/j.cub.2013.01.053

Duvarci, S., Nader, K., & LeDoux, J. E. (2005). Activation of extracellular signal-regulated kinase- mitogen-activated protein kinase cascade in the amygdala is required for memory reconsolidation of auditory fear conditioning. *European Journal of Neuroscience, 21*(1), 283-289. doi: 10.1111/j.1460-9568.2004.03824.x

Finnie, P. S., & Nader, K. (2012). The role of metaplasticity mechanisms in regulating memory destabilization and reconsolidation. *Neuroscience and Biobehavioral Reviews, 36*(7), 1667-1707. doi: 10.1016/j.neubiorev.2012.03.008

Jarome, T. J., Werner, C. T., Kwapis, J. L., & Helmstetter, F. J. (2011). Activity dependent protein degradation is critical for the formation and stability of fear memory in the amygdala. *PloS One, 6*(9), e24349. doi: 10.1371/journal.pone.0024349

Nader, K., Schafe, G. E., & Le Doux, J. E. (2000). Fear memories require protein synthesis in the amygdala for reconsolidation after retrieval. *Nature, 406*(6797), 722-726. doi: 10.1038/35021052

Reichelt, A. C., & Lee, J. L. (2013). Memory reconsolidation in aversive and appetitive settings. *Frontiers in Behavioral Neuroscience, 7*, 118. doi: 10.3389/fnbeh.2013.00118

Wang, S. H., de Oliveira Alvares, L., & Nader, K. (2009). Cellular and systems mechanisms of memory strength as a constraint on auditory fear reconsolidation. *Nature Neuroscience, 12*(7), 905-912. doi: 10.1038/nn.2350
