# Supplementary material for: Lack of drug-induced post-retrieval amnesia for auditory fear memories in rats
Source: BMC Biol. 2021 Jan 26;19:17. doi: 10.1186/s12915-021-00957-x (PMC7836479; doi:10.1186/s12915-021-00957-x)
Supplement: Supplementary file 3 — Additional file 3. Contains an extra experiment that was performed prior to and for the selection of the behavioral procedure for Experiment 7. [file 12915_2021_957_MOESM3_ESM.pdf]

Additional file 3

Experiment S7: Selection of the behavioral procedure for Experiment 7

In Experiment 7, we aimed to evaluate the amnestic effects of two frequently used protein synthesis inhibitors: anisomycin and cycloheximide. Although often employed to interfere with fear memories, we did not find published accounts of systemic administration of these drugs in a cued fear conditioning procedure in rats. As mentioned in the main text, both drugs have been applied systemically in several reports, and have been shown to produce amnesia in various behavioral procedures (e.g., Bernardi et al. 2007; Flint et al. 2007; Haubrich et al. 2015; Milekic & Alberini 2002; Taubenfeld et al. 2001; Wu et al. 2007), but have not yet been investigated in cued fear conditioning. In order to increase our chances of finding an amnestic effect, we therefore looked for papers with a cued fear conditioning procedure and local (intra-amygdala) infusion of these drugs, and conducted a dry run with these procedures (i.e., Experiment S7, vehicle animals only), with the purpose of selecting a behavioral protocol with similar freezing behavior in our hands as in the publications.

In a group of 8 animals, we aimed to replicate the procedure used by Duvarci et al. (2005, Fig. 1) as well as Nader et al. (2000, Fig. 5), a protocol that allowed them to induce post-retrieval amnesia with cycloheximide and anisomycin infusion, respectively. In what follows, this procedure is referred to as the Duvarci 2005 protocol. In a concurrently tested set of 8 other animals, we used the procedure of Nader et al. (2000, Fig. 2c, referred to as the Nader 2000 protocol), which also provided evidence for post-retrieval amnesia after anisomycin infusion, but did not feature a short-term memory test in between the Reactivation session and the long-term memory retention test (Test 1). We preferred a behavioral procedure that did include such a short-term memory (STM) test, because it would provide more information about the nature of the drug effects, if any.

Note that the vehicle used in this supplementary Experiment S7 was 60% DMSO (3 ml/kg DMSO and 2 ml/kg saline), which we intended to use in Experiment 7, but ultimately could not, because anisomycin did not dissolve, in contrast with Sigma-Aldrich’s product information.

Timing of the sessions and stimuli (***Table S1***) was the same as what was eventually used in Experiment 7, except that the exact replication of the Nader 2000 protocol in Experiment S7 did not include a Habituation phase nor Test STM.

Table S1: Parameters of conditioned (CS) and unconditioned stimuli (US) in Experiment S7.

Experiment number (experiment code on OSF)	CS (frequency, sound level, duration)			US (intensity, duration)		CS-US pairings during Training
Experiment S7 (Duvarci 2005 protocol) (LLERC16)	5000 Hz	80 dB	30 s	1.5 mA	1.0 s	1
Experiment S7 (Nader 2000 protocol) (LLERC16)	5000 Hz	75 dB	30 s	2.0 mA	1.0 s	1

Both behavioral procedures (***Fig. S1***) seemed to replicate quite well in our hands, and rats showed relatively limited between-subject variability in freezing. Given the added value of a short-term memory test, where we expected no amnestic effects of the drugs, we selected the Duvarci 2005 protocol (shown in the top panels of ***Fig. S1***) to be used in Experiment 7.

Mean freezing levels during the Reactivation CS, Test STM (average of CS1-3) and Test 1 (average of CS1-3) were overall similar, but somewhat higher in our hands (Experiment S7, Duvarci 2005 protocol: 56%, 75% and 71%, respectively) than in the control animals of prior publications (Nader et al. 2000: 66%, 54% and 56%, Duvarci et al. 2005: 43%, 44% and 44%, respectively, values obtained from graphs). Such generally higher freezing levels in non-operated animals are not uncommon, although it is not clear whether they reflect differences in fear or differences in freezing tendencies (Luyten et al. 2016; Zhang et al. 2001). Regardless, our data do indicate clear retrieval of the fear memory, no extinction resulting from the short-term memory test and sufficient room to observe amnesia in Experiment 7.

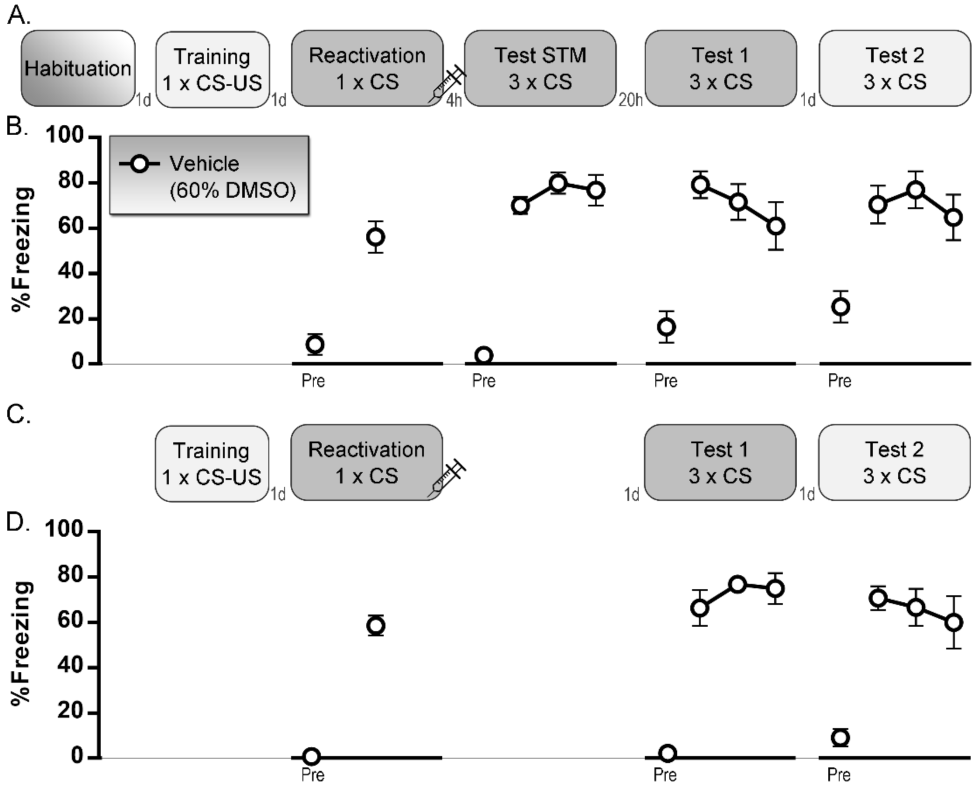

Figure S1: Experiment S7. A-B. *Duvarci 2005 protocol, n = 8 vehicle rats.* ***C-D.*** *Nader 2000 protocol, n = 8 vehicle rats. Percentage freezing during each tone (mean and SEM) is shown. 'Pre' is contextual freezing before the first tone presentation of the session. Light gray box indicates that a session takes place in context A, dark gray is context B. STM: short-term memory, CS: conditioned stimulus, US: unconditioned stimulus, DMSO: dimethyl sulfoxide, d: day, h: hours.*

References

Bernardi, R. E., Lattal, K. M., & Berger, S. P. (2007). Anisomycin disrupts a contextual memory following reactivation in a cocaine-induced locomotor activity paradigm. *Behavioral Neuroscience, 121*(1), 156-163. doi: 10.1037/0735-7044.121.1.156

Duvarci, S., Nader, K., & LeDoux, J. E. (2005). Activation of extracellular signal-regulated kinase- mitogen-activated protein kinase cascade in the amygdala is required for memory reconsolidation of auditory fear conditioning. *European Journal of Neuroscience, 21*(1), 283-289. doi: 10.1111/j.1460-9568.2004.03824.x

Flint, R. W., Jr., Valentine, S., & Papandrea, D., Jr. (2007). Reconsolidation of a long-term spatial memory is impaired by cycloheximide when reactivated with a contextual latent learning trial in male and female rats. *Neuroscience, 148*(4), 833-844. doi: 10.1016/j.neuroscience.2007.07.022

Haubrich, J., Crestani, A. P., Cassini, L. F., Santana, F., Sierra, R. O., Alvares Lde, O., & Quillfeldt, J. A. (2015). Reconsolidation allows fear memory to be updated to a less aversive level through the incorporation of appetitive information. *Neuropsychopharmacology, 40*(2), 315-326. doi: 10.1038/npp.2014.174

Luyten, L., Schroyens, N., Luyck, K., Fanselow, M. S., & Beckers, T. (2016). No effect of glucose administration in a novel contextual fear generalization protocol in rats. *Translational Psychiatry, 6*(9), e903. doi: 10.1038/tp.2016.183

Milekic, M. H., & Alberini, C. M. (2002). Temporally graded requirement for protein synthesis following memory reactivation. *Neuron, 36*(3), 521-525. doi: 10.1016/s0896-6273(02)00976-5

Nader, K., Schafe, G. E., & Le Doux, J. E. (2000). Fear memories require protein synthesis in the amygdala for reconsolidation after retrieval. *Nature, 406*(6797), 722-726. doi: 10.1038/35021052

Taubenfeld, S. M., Milekic, M. H., Monti, B., & Alberini, C. M. (2001). The consolidation of new but not reactivated memory requires hippocampal C/EBPbeta. *Nature Neuroscience, 4*(8), 813-818. doi: 10.1038/90520

Wu, C. R., Lin, L. W., Wang, W. H., & Hsieh, M. T. (2007). The ameliorating effects of LiuWei Dihuang Wang on cycloheximide-induced impairment of passive avoidance performance in rats. *Journal of Ethnopharmacology, 113*(1), 79-84. doi: 10.1016/j.jep.2007.05.003

Zhang, W. N., Bast, T., & Feldon, J. (2001). The ventral hippocampus and fear conditioning in rats: different anterograde amnesias of fear after infusion of N-methyl-D-aspartate or its noncompetitive antagonist MK-801 into the ventral hippocampus. *Behavioural Brain Research, 126*(1-2), 159-174. doi: 10.1016/s0166-4328(01)00256-x
